# Laparoscopic nephrectomy outside gerota fascia and en bloc ligation of the renal hilum for management of inflammatory renal diseases

**DOI:** 10.1590/S1677-5538.IBJU.2017.0363

**Published:** 2018

**Authors:** Liang Ma, Yanlan Yu, Guangju Ge, Gonghui Li

**Affiliations:** 1 Department of Urology, Sir Run Run Shaw Hospital, Zhejiang University School of Medicine, Hangzhou 310016, China

**Keywords:** Kidney Diseases, Laparoscopy, Nephrectomy

## Abstract

**Objectives:**

This study aims to improve laparoscopic nephrectomy techniques for inflammatory renal diseases (IRD) and to reduce complications.

**Materials and Methods:**

Thirty-three patients underwent laparoscopic nephrectomy for IRD, with a method of outside Gerota fascia dissection and en-bloc ligation and division of the renal pedicle. Operative time, blood loss, complications, analgesia requirement, post-operative recovery of intestinal function and hospital stay were recorded. The degrees of perinephric adhesion were classified based on the observation during operation and post-operative dissection of the specimen, and the association of different types of adhesion with the difficulty of the procedures was examined.

**Results:**

Among 33 cases, three were converted to hand-assisted laparoscopy, and one was converted to open surgery. Mean operative time was 99.6±29.2min, and blood loss was 75.2±83.5 mL. Postoperative recovery time of intestinal function was 1.6±0.7 days and average hospital stay was 4.8±1.4 days. By classification and comparison of the perinephric adhesions, whether inflammation extending beyond Gerota fascia or involving renal hilum was found to be not only an important factor influencing the operative time and blood loss, but also the main reason for conversion to hand-assisted laparoscopy or open surgery.

**Conclusions:**

In laparoscopic nephrectomy, outside Gerota fascia dissection of the kidney and en-bloc ligation of the renal pedicle using EndoGIA could reduce the difficulty of procedure and operative time, with satisfactory safety and reliability. Inflammation and adhesion extending beyond Gerota fascia or involving renal hilum is an important predictor of the difficulty related to laparoscopic nephrectomy for IRD.

## INTRODUCTION

Since laparoscopic nephrectomy was initially described by Clayman in 1991 (1), it has gained popularity for the treatment of a variety of benign and malignant kidney diseases. Particularly, for removal of kidney, in clinical practice, laparoscopic approach has gradually substituted open approach (2). However, in some cases with infectious and inflammatory kidney diseases, such as renal tuberculosis, pyonephrosis, pyelonephritis and xanthogranulomatous pyelonephritis, certain technical issues merit consideration while performing laparoscopic nephrectomy due to perinephric and perihilar adhesions, adhesions between the inflamed kidney and overlying bowel, and others. In such situations, laparoscopic nephrectomy was considered to be a challenging procedure for association with more complications, high probability of conversion to open surgery, bleeding, as well as injury to adjacent organ or large vessels. Thus, laparoscopic nephrectomy was regarded as relative contraindication under such conditions, and open approach was considered the procedure of choice (3). Nevertheless, disadvantages of open surgery are well known, including wider incision, more analgesics requirement, greater post-operative discomfort and pain, prolonged hospital stay, as well as longer convalescence period. Some urologists proposed that hand-assisted laparoscopic nephrectomy could help dissection of kidney and control of renal hilum, so as to reduce complications and shorten operative time (4-6). However, the incision is similarly longer and the injury is greater in this technique. Meanwhile, some other urologists attempted to perform nephrectomy to treat inflammatory kidney diseases entirely by laparoscopy and modified operation skills; nonetheless, the operative time was still long and with a certain likelihood of conversion to open approach (7, 8). In the present article, we introduced our clinical experience with an optimized procedure and the use of EndoGIA in laparoscopic nephrectomy for the management of inflammatory kidney diseases. To avoid the difficult procedures in laparoscopic nephrectomy with perinephric adhesion and hard handling of renal hilum, we applied a simultaneous method dividing of the renal hilum and resection of the retroperitoneal tissue with kidney en bloc during laparoscopic nephrectomy to excise the kidney. As far as we known, it is also the first time that we classified the degrees of perinephric adhesions and based on which we predicted the difficulty related to laparoscopic nephrectomy for IRDs and optimized the procedures of nephrectomy.

## MATERIALS AND METHODS

### Subjects

After institutional review board approval, data from a total of 33 patients undergoing laparoscopic nephrectomy for inflammatory kidney diseases at our hospital between June 2009 and August 2015 were collected. The patients aged between 27 and 76 years, with an average of 49.5 years. In this series, 14 patients were male and 19 were female, with 15 cases on the right side and 18 cases on the left side. All subjects presented with repeated lumbar pain preoperatively. 17 cases were diagnosed with chronic pyelonephritis (12 cases concomitant with kidney stones), 8 cases with renal tuberculosis, 4 cases of pyonephrosis, 3 cases of xanthogranulomatous pyelonephritis (XGP) and 1 case was a reno-colic fistula with infection. Prior to surgery, all affected kidneys were verified to be non-functional or severely damaged by ultrasonography, CT contrast imaging or radionuclide imaging of renal function. The patients with renal tuberculosis received anti-tuberculosis medication for at least 2 weeks before surgery. All operations were performed by a single urologic surgeon.

#### Surgical technique

**Figure 1:**
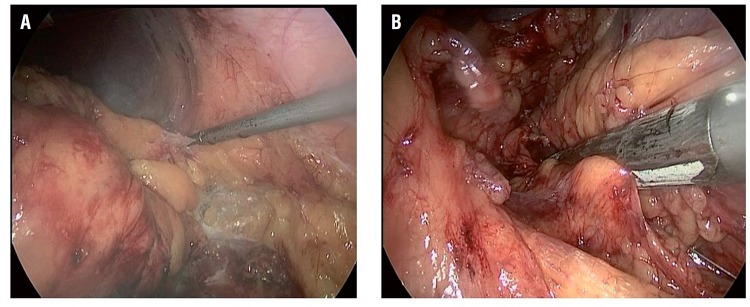
Laparoscopic procedure of nephrectomy for IBD. **A =** the kidney with its perirenal fat was dissected outside the Gerota fascia; **B =** the renal pedicle was divided and ligated by EndoGIA

The transperitoneal laparoscopic approach was applied for all the patients. Under general anesthesia, patients were positioned in a 70-90-degree supine-oblique position. A Veress needle was inserted in the paraumbilical region, and pneumoperitoneum was established with carbon dioxide insufflation to a maximum pressure of 15 mmHg. Three trocars were introduced: a 10-mm trocar for the camera in the paraumbilical region, two others (12 mm) for the surgeon's hands, one in the midclavicular line and the other one at the level of the umbilicus in the anterior axillary line. In some cases, additional 5-mm trocar in the midaxillary was placed for the assistant. Following inspection of the abdominal viscera, then, briefly, the peritoneum lateral to the colon was incised, the colon was reflected medially and the retroperitoneal space was sufficiently exposed. After inspecting the retroperitoneal adhesion, the kidney with its perirenal fat was dissected outside the Gerota fascia, similar to the procedure of radical nephrectomy: dissection of the lower pole of kidney together with the adipose capsule to further expose the psoas muscle, then lifting the lower pole of kidney and dissecting proximally to the lower edge of the renal hilum. Subsequently, Gerota fascia was incised from the upper pole of kidney and above the hilum, preserving the adrenal gland. The dissection continued posteriorly to the psoas muscle and further inferiorly to the upper edge of renal hilum. Along the surface of the psoas fascia, dissection continued from the lower edge of the renal hilum posteriorly to the upper edge of the hilum, exposing the renal pedicle completely. With an endovascular gastrointestinal anastomosis (EndoGIA), the renal pedicle was divided (Figure-1). After identification of the ureter in the lower region of the surgical field, it was occluded with Hem-O-Lok vascular clip (Weck; Telefex Medical, Durham, NC, USA) and then divided; for renal tuberculosis, the ureter was isolated downwards until it became soft. Thus, the Gerota fascia was left intact, and the kidney accompanied with its adipose capsule was removed en bloc. The specimen was placed in a surgical bag refraining from contamination of the peritoneal cavity. Then a 4-7 cm lateral rectus incision was made through which the bag was removed. A tube drain was brought out through one incision.

Intraoperative events, operative time, blood loss, and injury to adjacent organs were recorded. The degrees of perinephric adhesions were evaluated by dissection of the specimens along with intraoperative findings. Postoperative complications were also recorded. All patients were followed up at 2 weeks, 3 months and 6 months postoperatively.

#### Statistical analysis

Results are expressed as mean ± standard deviation, unless otherwise indicated. Statistical significance between two groups was determined by two-tailed Student's t test. SPSS 16.0 package program was used (SPSS Inc, Chicago, Illinois, USA) and P<0.05 was considered statistically significant.

## RESULTS

In all thirty-three cases, three were converted to hand-assisted laparoscopy and one was converted to open surgery. The operative times ranged between 64 and 217 minutes, with a mean of 99.6±29.2 min. The mean blood loss was 99.6±29.2 mL, with a range of 20 to 500 mL. The mean recovery time of intestinal function postoperatively was 1.6±0.7 days (range 0.5 day to 5 days). The length of hospital stays after surgery ranged between 3 and 11 days, with an average of 4.8±1.4 days.

Conversion to hand-assisted laparoscopy was conducted in 3 patients, because it was too difficult to expose the renal pedicle, as well as hilar lymphadenopathy. In these cases, Hem-O-Lok clippers were used to clip the renal artery and renal vein respectively, with 2 clippers placing in the proximal side and 1 clipper in the distal side, and then divided. For these three cases, the mean operative time was 120 minutes, with an average blood loss of 117 mL and an average postoperative hospital stay of 4.7 days. Open conversion occurred in one patient with fistula between the left kidney and descending colon. The kidney was removed and the descending colon was repaired. The operation lasted for 217 minutes, with blood loss of 500 mL and postoperative hospital stay of 11 days.

Two patients developed fever (>38.5°C) postoperatively, and their symptoms relieved after appropriate antibiotic treatment. One patient had slow recovery of intestinal function whose bowel sounds occurred at postoperative day 4 and had passage of gas by anus at day 5. In addition, one patient had incisional infection and recovered after adequate dressing and drainage. All the patients returned to normal life and work without severe complications within 3 months after the surgery.

Extent of perinephric adhesion was evaluated by intraoperative findings and dissection of the specimen. The characteristics of different degrees of perinephric adhesion are summarized in Table-1 Inflammation involving adipose capsule and not extending into the renal hilum in 17 cases (51.5%) (B+C), involving the renal hilum and/or beyond the Gerota fascia in 13 cases (39.4%) (D) and invading adjacent organs in 3 cases (9.1%). Operative time and blood loss were not significant different between B1, B2 and C groups, but significantly increased in D and E groups. Compared with B+C groups, the operative time in D group prolonged significantly (P=0.007, P<0.01), and blood loss in D group was significantly higher (P=0.005, P<0.01). The results demonstrated that by applying our operative technique, adhesion involving the renal pedicle or tissues outside Gerota fascia was the major factor associated with operative time, blood loss, and conversion to hand-assisted laparoscopy or open surgery.

**Table 1:** Clinical features of the patients with different degrees of perinephric adhesion.

Degrees of perinephric adhesion	N	Conversion to hand assistance	Conversion to open surgery	Operative time (min)	Blood loss (mL)	Postoperative hospital stay (days)	Complications
A	0						
B B1	5			82.2±15.5	37.0±9.7	4.6±0.5	
B2	7			78.7±8.84	44.3±7.9	4.3±0.5	One had a fever
C	5			83.6±6.9	41.0±15.2	4.4±0.9	One had delayed recovery of intestinal function
D	13	2		110.4±14.6	83.1±34.1	4.8±1.0	One had a fever and another one had infection of the incision
E	3	1	1	157.3±53.2	233.3±236.3	7.0±3.5	
B+C	17	0	0	81.2±10.3	41.2±10.7	4.4±0.6	2
**Total**	**33**	**3**	**1**	**99.6± 29.2**	**75.2±83.5**	**4.8±1.4**	**4**

**A =** The degrees of perinephric adhesion were without involvement of the adipose capsule; **B =** involvement of the adipose capsule but not extending into the Gerota fascia or renal hilum; **B1 =** <1/2 adipose capsule and B2-≥1/2 adipose capsule; **C =** involvement of the Gerota fascia but not extending into the renal hilum; **D =** involvement of the renal hilum and/or beyond the Gerota fascia but not invading adjacent organ; **E =** invading adjacent organs.

## DISCUSSION

At present, the major causes for kidney resection are still benign kidney diseases (9). Rassweleir et al. (3) reported that in a series of 482 nephrectomy operations, 92% were due to benign kidney diseases. Patients with chronically non-functioning symptomatic inflammatory kidney diseases usually necessitate surgical removal of kidneys, especially when they present with severe lumbar pain, recurrent urinary infection or renovascular hypertension. However, the best approach is still controversial because the nephrectomy for IRD is highly challenging concomitant with considerable complications. The difficulty for operation arises from the dense adhesion between the kidney and adipose capsule or Gerota fascia, perihilar adhesion or fibrosis, as well as the inflammation involving the adjacent structures such as the liver, spleen, intestine, psoas muscle, diaphragm or spinal column.

Earlier investigators were of the opinion that open surgery was faster and less prone to complications than laparoscopy (10) with easier dissection of the kidney and better exposure of the adjacent structures (11). Moreover, they did not advocate laparoscopic nephrectomy because of adhesions and the risk of abdominal contamination with purulent exudate (12), and believed that inflammatory kidneys were responsible for higher rate of complications and conversions to open surgery (3, 13).

With the accumulation of clinical experience and development of laparoscopic techniques, more and more urologists conduct laparoscopic nephrectomy for IRD. Khaira and associates (14) found that the incidence of complications between laparoscopic and open techniques is comparable, and, when laparoscopic nephrectomy was feasible, patients have the benefit of a minimally invasive procedure. More recently, Vanderbrink (15) described his experience comparing open versus laparoscopic nephrectomy in patients with XGP and concluded that there was no statistical difference regarding blood loss, transfusion rate, or parenteral analgesic requirements between both groups; in addition, there was a trend toward a shorter stay for the laparoscopic group. Manohar et al. (7) and Hatipolu et al. (16) reported that the post-operative hospital stay of laparoscopic nephrectomy and open surgery was 1.5 (1-7) days, 3.5 (3-5) days respectively, which were significantly shorter than open surgery.

Increasing clinical studies demonstrated that the operative duration for laparoscopic nephrectomy for IRD decreased gradually, with a trend for lower rates of conversion to open surgery and complications (Table-2) (7, 8, 17-22). We considered that it was associated with the maturation of urologists’ skill, as well as doctors’ better understanding of the nature of the diseases and selecting more amenable patients to perform laparoscopy.

**Table 2:** Comparison of intraoperative and postoperative data in literatures on series of laparoscopic nephrectomy for inflammatory diseases.

References	N	Access	Mean operative time(min)	C	Complications	Discharge from hospital(days)
Shekarriz et al. (16), 2001	12	TP	284	2	0	4.1
Lee et al. (17), 2002	21	RP	244	RP1	0	5.3
	10	TP		TP1		
Tobias-Machado M et al. (18), 2005	11	RP	160	0	2	3
	6	TP(HA)	200	2	2	4.3
Manohar T et al. (6), 2007	84	TP	170	8	26	4.34
Duarte RJ et al. (7), 2008	50	TP	194.2	14	6	8.34
Guzzo TJ et al.(19), 2009	14(XGP)	TP	228	1	6	3
Marcelo Lima et al. (20), 2012	66(XGP)	TP	122.5	8	3	2.1
Kaba M et al. (21), 2015	15 with stones	TP	95.0	1	1	2.93
	17 without stones		86.7			2.59
Present study	33	TP	99.6	1	4	4.8

**RP =** retroperitoneal; **TP =** transperitoneal; **HA =** hand assistance; **C =** conversion to open surgery; **XGP =** xanthogranulomatous pyelonephritis

Currently, laparoscopic approaches to perform kidney operation include transperitoneal and retroperitoneal routes. The transperitoneal approach offers a larger working space, readily identifiable anatomic landmarks and greater distance between the trocars so as to facilitate the urologists to maneuver more easily. The retroperitoneal approach reduces interference with the peritoneal cavity and is preferred particularly in patients with prior abdominal surgery. Some studies concluded that the operative duration and post-operational hospital stay for retroperitoneal nephrectomy were shorter (23). Nevertheless, in clinical practice, urologists prefer to conduct transperitoneal approach for patients with large-sized kidney or dense adhesions, or even choose hand-assisted laparoscopy, which might be the reason for the worse outcome of transperitoneal route compared with retroperitoneal route (19).

In this study, we evaluated the extent of perinephric adhesion, perihilar adhesion and involvement of adjacent organs by dissection of the specimen along with intraoperative findings and classified the degrees of adhesion. We noted that there was no statistically significant difference in terms of operative time or blood loss between the groups in which inflammation was confined within the Gerota fascia. However, when inflammation involved the renal hilum, beyond the Gerota fascia or invading adjacent organs, the operative time prolonged and blood loss increased, and there was a statistically significant difference compared with that within the Gerota fascia. Moreover, the rate of conversion to open or hand-assisted laparoscopic procedures were higher. Consequently, we believe that inflammation extending beyond Gerota fascia or involving renal hilum is an important predictor of the difficulty related to laparoscopic nephrectomy for IRD.

Dissection of the kidney is a difficult step in the laparoscopic nephrectomy for IRD. Kapoor et al. (24) chose subcapsular dissection in most of their patients and had an 80% success rate in patients with XGP, thus they concluded that fewer complications occurred compared with open surgery. Xu et al. (25) also considered the subcapsular laparoscopic nephrectomy as a safe way to remove an infected and tightly adherent nonfunctioning kidney using the retroperitoneal approach.

To avoid dissecting the perinephric adhesion, we applied the outside Gerota fascia approach the same as in radical nephrectomy, with satisfactory results. This result was in accordance with that of other researchers and further confirmed the feasibility and safety of outside Gerota fascia dissection.

Dissection in the renal hilar region is another difficult step of laparoscopic surgery. Some investigators considered hilar dissection the most challenging step because of the risk of hemorrhage. In our experience, we used EndoGIA to en-bloc ligate and divide the renal pedicle following dissection of the renal hilum to a certain degree, averting fine separation of the renal artery, vein and lymphatic vessels in this area, therefore the difficulty of operation, and blood loss decreased and time was saved. No associated complications including arteriovenous fistula were observed during the operation and follow-up. Chung et al. (26) and Rybak et al. (27) have reported their experiences of handling the renal pedicles with en-bloc stapler ligation in laparoscopic radical nephrectomy and laparoscopic nephrectomy. Their results revealed that this method is safe, reliable and simple; and they reinforced that there was no evidence of arteriovenous fistula formation. However, under certain circumstances such as the presence of many lymph nodes around the pedicle and tissues very thick and hard, which makes the stapler difficult to close, we have to dissect the renal vein and artery carefully and separately.

In our series, two cases were converted to hand-assisted laparoscopy due to the significant hilar fibrosis and failure to use EndoGIA. One case was converted to hand-assisted technique due to tight adhesion between the kidney and ascending colon in addition to severe hilar adhesion. And one case was converted to open surgery for fistula between the lower pole of kidney and the descending colon. The result suggested that in patients with particularly severe inflammation involving the renal hilum or invading the adjacent organ, conversion to hand-assisted or open surgery is inevitable to facilitate completion of the operation.

## CONCLUSIONS

As experience grows and instrumentation improves through the combined collaborative efforts of surgeons and medical technologists, laparoscopic nephrectomy for IRD is gradually becoming safe and feasible. Outside Gerota fascia dissection of the kidney and en-bloc ligation and division of the renal pedicle by using EndoGIA could reduce the difficulty of procedure and operative time, with satisfactory safety and reliability. Inflammation and adhesion extending beyond Gerota fascia or involving renal hilum is an important predictor of the difficulty related to laparoscopic nephrectomy for IRD.
